# An Investigation of the Theories of the Natural History of Man, by Lawrence, Prichard, and Others; Founded upon Animal Analogies; and an Outline of a New Natural History of Man; Founded upon History, Anatomy, Physiology and Human Analogies

**Published:** 1848-07

**Authors:** 


### BIBLIOGRAPHICAL NOTICES.

*An Investigation of the Theories of the Natural History of Man, by Lawrence, Prichard, and others ; founded upon animal analogies ; and an outline of a new Natural History of Man ; founded upon History, Anatomy, Physiology, and Human Analogies.* By FREDERICK VAN AMRINGE. New York. 1848. pp. 739. 8vo.

We sat down with the intention of examining this work for the purpose of critical review. A few hours of study convinced us that this was impossible, within the space and time allowed for a contribution to a medical journal ; so vast and various is the mass of materials which it contains. Our next intention was to give a terse analysis of its contents, without any expression of opinion as to the fairness and logical merit of the argument, or the justice of the conclusions : but this appeared a dereliction of duty, both to the public and the author, and we are driven upon the only remaining alternative, a rapid sketch of a few of the principal problems involved, with a running, and, it may be, a rambling commentary ; sufficient, however, to direct the attention of the learned and literary among our readers, to this very remarkable labour of an American author.

The work, as we are told in the preface, was written at a distance from capitals and libraries, "in an agricultural district," and while the author was in ill health; so that it is reasonable to infer that it was also produced in comparative haste. This is much to be regretted, for although it constitutes, under such circumstances, a monument of extensive reading and wonderful industry, the field of discussion is too broad to be accurately and safely traversed, even by the brightest mind, aided by every facility for research, in less than several years. Had the author been more favoured in point of time and opportunity, it can scarcely be doubted that many defects would have been removed by the pruning knife.

The main purpose or end of the entire work, is the establishment of the proposition that man, the genus, is zoologically divisible into at least four distinct species. So strong is the internal testimony, on almost every page, that this was a preconceived opinion, that we presume the author will exonorate us from all unfairness in regarding it as the guiding star of his labours throughout, although we are informed that his intention was originally confined to a review of the reasonings of Lawrence and Prichard, on the natural history of man, as founded upon animal analogies.

In approaching the historical branch of the subject, he is met, at once, by that stumbling-block in the way of so many scientific men, the Mosaic history, and, at a later period, by the account given of the distribution of mankind by Josephus. He declares his unhesitating faith in the entire record of revealed religion, even inclining to the strict literal acceptation of the six days of creation. But, though evidently an advocate of the Mosaic history and the Christian morals, our author, in an introductory chapter, exerts much ingenuity in freeing the path of philosophical research from all restraints not directly deducible from the text of scripture. Thus, he just hints, and immediately relinquishes as unnecessary to his argument, a variety of curious hypotheses, more or less original, which may be valuable hereafter, to the poet at least, if not to the naturalist; among which the principal are a shadowy, typical parallelism between certain stages in the vital history of the preadamite ages, and certain epochs in the developement of man, as a psychical being; such as the

Adamic, the Noachian, and the Christian periods; also, the possible existence of other human inhabitants of the earth, at the time of the Adamic creation, and the possibly limited character of the Noachian deluge; all which questions, he thinks, may be mooted even under the literal version of the Mosaic history of the six days.

Adhering, however, on full conviction, to the biblical account of the deluge, he acknowledges, in the face of his peculiar opinions as to the *existing* specific distinctions among mankind, that all human inhabitants now living upon earth are ultimate descendants of one common male progenitor, Noah. Nor does he attempt to trace the different races or species of man, through the female line, into the ante-diluvian period. Employing the discoveries of paintings in Egypt, *legitimately* to show the existence of the red, white, and black races at a period approaching to that of the deluge, he starts the hypothesis that the division of the human family into four species was the result of physical or psychical changes consequent upon the curse of the Almighty, through Noah, upon Canaan the oldest son of Ham; his blessings upon Shem and Japhet, spoken by the same patriarch; and, at a later period, the blessing, through the angel, on the offspring of Hagar, the Egyptian, and Abraham, the Shemite.

In preparing for the development of this hypothesis, the distribution of mankind becomes an element in the argument. The statements of Josephus, furnishing the chief historic basis of the prevailing doctrines in relation to this subject, interfered with the favourite idea of our author, that the actual superiority of the white race of mankind is a consequence of the superior blessing bestowed through Noah upon Shem, in whom all the earth was to be blest, and whose mission in the divine scheme passed from the white descendants of Abraham to the whites of Europe. Josephus supposed Europe to have been populated by the offspring of Japhet, and it became necessary to examine the foundations of the positions assumed by the Jewish historian. Mr. Van Amringe has done so; drawing the conclusions—first, that Europe was really populated by the descendants of Shem, with whom were intermingled the progeny of Ham, through all his sons, with the exception of the accursed Canaan, constituting the white, or Hamoshemetic species of man; second—that the yellow species is Japhetic, and includes the Chinese, Mongolian, Japanese, Chin Indian, and probably the Eskimautic, Toltec, Aztec and Peruvian varieties; third—that the red, or copper-coloured species, is Ishmaelitic, and includes most Tartar and Arabic tribes, and all the American Indians, not already mentioned; and finally, that the black species is Canaanitic, and includes the Central African and Australasian Negroes, Caffirs, Malays, &c.

This part of the argument labours to overthrow the doctrine of Josephus, and would leave our preconceived notions of the distribution of mankind without solid foundation, unless supported by other arguments derived from the traditions of barbarian ages or mythologic allegory. But are the positions of our author established by his reasonings? We doubt the issue of the argument with the learned, but would not undertake to express an opinion unless after years of research.

Mr. Van Amringe does not rest his argument on the diversity of the human species entirely upon this hypothesis of blessings and curses, or upon the history of the distribution of mankind after the dispersion of Babel; for he discusses at great length the purely zoological view of the subject, both with and without reference to the questions of a single centre of distribution, and the descent from a single original pair. Yet we cannot remove the impression that this hypothesis, the influence of which upon his mind is obvious in almost every part of the work, is one among the chief causes of an apparent disposition to look through an exaggerating medium upon the almost utter hopelessness of the mental condition of three of the races of man, and the vast intrinsic superiority of that race to which we have the happiness to belong.

This bias even leads him into some seeming incongruities. Thus; he observes that the budding civilization of mankind, prevalent on the plain of Shinar before the miraculous separation of the species, was shared to an equal degree by all the races at the time of the confusion of tongues; but, while the peculiarly favoured Hamoshemetic nations have steadily advanced in the indefinite improvement which is *a generic peculiarity of human nature*, the red and yellow species have remained stationary, after some slight improvement, while the Canaanitic nations have actually retrograded. The inference is drawn that the superior powers of the Hamoshemetic race were conferred by the Creator, for the purpose of making it the special recipient and missionary of light, intelligence and religion; and that even the *generic* tendency to indefinite improvement cannot be effectively developed without its agency! In one moment of peculiar despondency, our author almost despairs of the ultimate fate of the "servant of servants unto his brethren," unless redeemed by the operation of the American Colonization Society, and other rational efforts of this day; seeming to think that the very species is possibly destined to become gradually extinct, before the march of the whites, unless redeemed by the philanthropic exertions commenced in the present age.

While granting the important tendency of the labours of Mr. Van Amringe, in checking some of the most pernicious attempts of misguided benevolence to ameliorate the condition of inferior races by means totally inapplicable to their existing mental condtion—attempts which are much promoted by a hasty misconstruction of the beautiful Jeffersonian principle of liberty and *equality* in the Declaration of Independence, we regret that the writer seems to possess so little faith in his own *generic* character of indefinite improvement. We have somewhat higher faith in the internal capacities of entire humanity, while fully appreciating, as we believe, the true value of all the evidence in the work before us.

Mr. Van Arminge has given us a modified zoological classification of the *mammalia*, in order to place the genus *Homo*, and its subordinate species, in more precise relations, with animals in general and with each other.

The chief peculiarity of this arrangement is the attempt to separate man more widely from "the (rest of?) the animal kingdom, by means of his psychical nature." "Man," according to him, "is something more than an animal." Both have instincts, it is true; but these are treated, according to custom, as mere functions of the nervous system, while man has intellectual faculties; and we think we do no injustice to the author's argument, in stating that these are employed therein as though they were attributes of something beyond all functions of the nervous system, and, therefore, entirely transcendental to any property of an animal.

We should not follow him into these deep waters, if it were not that he employs in a novel and original manner, throughout his reasonings, this long acknowledged, but, as we hold it, over-estimated mental superiority of humanity. This furnishes him with weapons for severe and, sometimes, pleasantly sarcastic criticism upon Lawrence, Prichard, and others; much of which is just, while some of it appears to involve a *petitio principii.* It leads him to the condemnation of all *animal analogies*, in reasoning upon the question of specific distinctions among mankind; yet, strange as it may appear, he thinks proper to resort largely to such analogies in strengthening his argument upon the tendencies of hybrid races to return to the original stock; which, if not established, might weaken the support of the doctrine of the miraculous division of species. Upon this total separation of man from the animal creation, depends much that is urged against the power of climate, food, domesticity and other accidents in connection with time, to produce such long enduring distinctions as are observable in the human family. We shall not contend that his conclusions would entirely fall to the ground on the overthrow of this psychical doctrine, in which most men, with the exception of the materialist among the phrenologists, will probably agree with him, unless the gift of speech should be, at some future time, conferred upon animals in general, who are certainly parties interested in the case. But we do contend that this doctrine, which is interwoven with the whole argument, and used as an axiom, is a mere postulate, and one which we are by no means disposed to grant. If ever we should be employed as counsel in the case, we shall be happy to measure philosophical swords with the author on this great question.

We believe that *mind* is a property of "the brutes that perish," as well as of man: that perception is a purely psychical phenomenon; that instinct is associated as closely with perception as is the intellect; that the only routes by which knowledge can be acquired by a psychical being in this state of existence, are—*direct revelation* from the Creator, and *sensation*, a function of the nervous system—by which latter route the *written* revelation reaches man, and *may be* aided by direct revelation when the mind is directed to the text of scripture by the will; that the truly fundamental psychical phenomena are *consciousness* and *will;* the first only arousable by the functional action of certain nerves or by revelation, and the second reacting upon certain nerves, whether to produce voluntary motion or those complex psyco-physiological operations of consciousness called judgment, conscientiousness, imagination, amativeness, wit, combativeness, causality, &c.—be they artificially divided into instinctive, moral and intellectual, or viewed in the totality, as *the mind.* Finally, we hold that there exists no such thing as psychical science or proper meta-physics. In all other things than consciousness and will, (the latter of which we know to exist solely by means of the former,) the mind, or, if the reader please, *the soul,* acts only upon exterior objects and is acted upon only by such objects, through the media of the nerves or by direct revelation. Not being exterior to itself, the mind or soul cannot observe itself or reason upon itself, but is simply conscious of itself, and inclined, withal, to be very wilful, especially in argument.

Now, as questions of direct revelation lie beyond the domain of reason; and as the written revelation is subject to the action of senses; it follows that all philosophical investigations are limited by the operation of *senses,* and governed most absolutely by the laws of the nerves of animal life and purely animal physiology: they are in no degree psychical in their nature, though they can only be pursued by psychical beings. If there be any indisputable fact positively proving the absence of any *specific* power of the mind in any psychical being, with or without brains, from the germ of a zoophyte up to "Bacon and Newton," we have failed to discover it, during some considerable number of years mainly devoted to physiological thought and study. The young mammal might turn to the mamma, when present, by a nervous agency, independent of mind; but even when habituated to its sweets, it would never wander in search of the mother, when hungry, unless *because* it is a psychical being:—otherwise, its motions would be quite as involuntary as those of a tortoise, after the head with the *medulla oblongata* are removed.

Much of what has been written in proof of the vast hiatus between man and the rest of the animal creation is subject to the just satire directed by our author against those who learnedly endeavour to prove that *man is not an ape:*

"Much labour," he remarks, "has been bestowed by philosophers to show that the erect attitude does not belong to monkeys, and particularly apes; which last have been particular objects of fear to them, lest they should become Scotch cousins."

Our author is playful: he will permit us to be so. Is it not possible that the closer approach between the coloured and white races may make the peculiarly favoured Shemitic portion of humanity quite as desirous of widening the breach? Let us not be misunderstood; we are no advocate of "universal fraternization;" taste would prohibit it, if physiological propriety did not. Nor do we deny the marked distinctions between the races which actually exist—they are quite as great as is desirable, but are they, as Mr. Van Amringe contends, *specific?*

The views just expressed will probably appear quite as startling and peculiar to many, as are those advanced by the writer under review, and he is entitled to all proper allowances for this fact, on the part of our readers; but we are inclined to think that candid and deliberate judges, in perusing the work immediately under notice, will agree with us in the opinion that the ultraism of the warm defender of an original position is perceptible in those parts of the argument with which the supposed value of the psychical distinctions of men are most closely involved. They will agree that he has been too severe in his opposition to animal analogies in illustrating the natural history of man, the animal; that he has under-estimated the value of governments, established customs, peculiar religions, climates, social institutions, (with the exception, perhaps, of those connected with the sexual relations,) and other accidents of somewhat permanent duration, in modifying for long periods the anatomical structure, and, consequently, the physiological and miscalled psychological phenomena of human nature, and that he has done so from the constant influence of one predominating idea. Yet in all these particulars the evil consequences of this prejudice (we use the word in its derivative sense only,) are far more important in their bearing upon the numerous *lemmas* eliminated in the course of the argument, than in their relation to the main problem of the specific distinctions of man, to which none of them are of vital consequence. In this sense, the consequent errors of opinion, if such they be, are errors of intensity and not of essential construction.

The same unconscious diminution of obstacles and enhancement of favouring facts, is obvious in the anatomical and physiological passages. The only decided originality of opinion observable in this department of the argument, consists in the unusual importance attached to the skin, and more especially the rete mucosum, with its sympathetic and structural relation to other parts of the human machine. The scarcely debateable point of the existence of this part as a true rete in the white race is decided in the negative, though its presence as a microscopic rudiment is mentioned, and the principal authorities quoted. The influence of the skin over the nervous system is pressed, we think, decidedly beyond its acknowledged importance, and the reasoning upon the agency of the elementary constituents of the black pigment in the chyle, the neurilema and the "flesh!" &c., are so far at war with established physiological laws, that we are compelled to condemn them.

Our author is somewhat of a phrenologist—that is, he acknowledges the location of the physical instruments of the intellectual, moral, and instinctive powers of the mind, in those regions of the cavity of the cranium pointed out by the phrenologists; but he believes this as a simple fact, drawn from observation, knowing of no *physiological* evidence in proof of its truth! Need we say that the writings of the fathers of Phrenology are full of such proofs? We could easily add to them were it necessary; but there is a vagueness about this part of the argument that induces regret that it was not subjected to more thorough digestion and deeper research. In fact, rigorous physiological reasoning does not appear to be the forte of the writer.

The importance of woman and the sexual relations in the history of civilization form the principal subjects of terminal chapters of the work; and this cannot be easily over-estimated, though the value of the social condition of the wife as a specific character of the races of man will undoubtedly be questioned.

We will conclude by stating that the volume is crowded with a multitude of details, drawn from a vast range of human knowledge, and is eminently suggestive of curious speculations. Morally, its highest usefulness will probably be found in its tendency to fix attention upon the very wide and durable—perhaps perdurable—distinctions actually existing between the races of man; and were the same class of researches cautiously pursued, through the national varieties, and even subvarieties of humanity, great practical benefits might result in matters of government, morals and benevolence: but after allowing all due weight to the facts and ingenious arguments adduced, we are still unprepared to regard the division of the human family into at least four distinct species as an indisputable zoological fact. * *

